# Cell-surface nucleolin acts as a central mediator for carcinogenic, anti-carcinogenic, and disease-related ligands

**DOI:** 10.1007/s00432-014-1587-5

**Published:** 2014-01-28

**Authors:** Hirota Fujiki, Tatsuro Watanabe, Masami Suganuma

**Affiliations:** Research Institute for Clinical Oncology, Saitama Cancer Center, Saitama, 362-0806 Japan

**Keywords:** Carcinogenic mediator, EMT, HB-19, Nucleolin, Tipα

## Abstract

**Purpose:**

Cell-surface nucleolin in human gastric cancer cell lines is a receptor for TNF-α-inducing protein (Tipα) of *Helicobacter pylori*. The binding complex of nucleolin and Tipα is internalized into the cells and then induces tumor progression of human gastric cancer. Surface nucleolin is also a receptor of human immunodeficiency virus-1, and the anti-HIV pseudopeptide (HB-19) showed anti-carcinogenic activity in vivo. Surface nucleolin has dual functions depending on the ligands: In order to understand the mechanisms of surface nucleolin, it is necessary to review surface nucleolin and its relation to carcinogenic ligands and anti-carcinogenic ligands. Other ligands can be grouped among disease-related ligands, which is an important new topic for the prevention of various ailments.

**Results and discussion:**

This paper mainly deals with two ligands of surface nucleolin, Tipα and pseudopeptide HB-19. The binding complex of nucleolin and Tipα induces expression of *TNF*-*α* and *chemokine* genes and activates NF-κB in gastric cancer cells of humans and mice. However, when human gastric cancer cell line MKN-1 was transfected with nucleolin-targeted siRNA, the result was inhibition of cell migration and elongation induced by Tipα. The amount of surface nucleolin was reduced in membrane fraction of the nucleolin knockdown MKN-1 cells, but the amount of nucleolin in the cytosol or nuclear fractions of the cells did not change. The results indicate that surface nucleolin acts as a carcinogenic mediator for Tipα of *H. pylori.* In contrast, both the viral external envelop glycoprotein gp120 of HIV and the anti-HIV pseudopeptide HB-19 bind to surface nucleolin. Through this binding, treatment with HB-19 inhibited tumor development in human breast cancer cell line MDA-MB-231 and rhabdoid tumor cell line derived from Wilms’s tumor in xenograft nude mouse models. The results show that surface nucleolin acts as an anti-carcinogenic mediator for HB-19.

**Conclusion:**

Based on these discrete functions of surface nucleolin, the binding complex of carcinogenic ligands and surface nucleolin seems to be competing with that of anti-carcinogenic ligands and surface nucleolin. Moreover, carcinogenic ligands derived from endogenous sources play a significant role in human cancer development, and the interaction of surface nucleolin with disease-related ligands will be a new research subject for the prevention and treatment of various ailments.

## Introduction

Nucleolin, with a molecular weight of 105–110 kDa, initially called C23, is originally one of the 100 distinct proteins: It makes 5–10 % of total nucleolar protein in normal rat liver and Novikoff hepatoma ascites cells (Orrick et al. 1973; Bugler et al. 1982). Human nucleolin cDNA, containing the entire coding region, was isolated from lambda gt110 human retinal library, and the human nucleolin gene consists of 14 exons and 13 introns on chromosome 2q12-qter (Srivastava et al. 1990). The translation of the nucleotide sequence predicted a 707-amino acid protein with several discrete domains (Srivastava et al. 1989), and amino acid sequence analysis of nucleolin revealed three structural domains: The N-terminal domain contains both highly acidic regions and multiple phosphorylation sites; the central domain contains four RNA-binding domains (RBD or RRM); and the C-terminal domain (RGG or GAR) is rich in glycine, arginine, and phenylalanine residues (Ginisty et al. 1999). Nucleolin was previously thought to be a simple RNA-binding protein involved in the organization of nucleolar chromatin, packaging of pre-RNA, rDNA transcription, and ribosome assembly by shuttling between nucleus and cytoplasm; recent investigation has further revealed that nucleolin is involved in modulating transcriptional process, cytokinesis, nucleogenesis, signal transduction, apoptosis, induction of chromatin decondensation, and replication (Srivastava and Pollard 1999).

Although more than 90 % of nucleolin is found in the nucleolus, phosphorylated nucleolin is found on the cell surface of logarithmically growing simian virus 40-transformed mouse fibroblasts (Pfeifle and Anderer 1983). Nucleolin has been classified into nuclear, cytoplasmic, and cell-surface nucleolins, depending on location in the cells; both nuclear and surface nucleolins are phosphorylated (Belenguer et al. 1989; Jordan et al. 1994). Nuclear nucleolin has pI values between pH 4.0 and pH 6.0, and surface/cytoplasmic nucleolin is about pH 4.5. Cytoplasmic nucleolin is found in small vesicles that appear to translocate nucleolin to the cell surface, and this translocation is temperature dependent. Nucleolin is expressed at the cell surface, where it exists in close association with intracellular actin cytoskeleton, and surface nucleolin is clustered at the external side of plasma membrane (Hovanessian et al. 2000). The half-life of nuclear nucleolin is estimated to be more than 8 h, while that of surface nucleolin is <1 h (Hovanessian et al. 2010). Surface nucleolin is recently attracting great attention as an important receptor of cells for numerous ligands derived from various sources.

It is of interest to note that surface nucleolin plays a critical role in carcinogenesis, since endogenous ligands such as midkine, pleiotrophin, P-selectin, and hepatocyte growth factor (HGF) are involved in carcinogenesis (Table 1) (Kadomatsu and Muramatsu 2004; Reyes-Reyes and Akiyama 2008; Tate et al. 2006). In our study on *Helicobacter pylori* (*H. pylori*) carcinogenesis, we discovered that tumor necrosis factor-α (TNF-α)-inducing protein (Tipα) of *H. pylori* is a carcinogenic factor that induces tumor promotion in vitro and in vivo (Suganuma et al. 2001, 2005). It was exciting for us to find that the exogenous ligand Tipα binds to surface nucleolin on human gastric cancer cell lines and that internalization of the Tipα and nucleolin complex induces tumor progression and epithelial–mesenchymal transition (EMT) in human gastric cancer (Suganuma et al. 2008; Watanabe et al. 2013): Our study showed that the binding of Tipα to surface nucleolins is an essential step for *H. pylori* carcinogenesis in humans. Hovanessian and his colleagues discovered that surface nucleolin serves as a low-affinity receptor for human immunodeficiency virus-1 (HIV-1), and they further demonstrated that treatment with anti-HIV pseudopeptide HB-19 inhibits tumor development of human breast cancer and rhabdoid tumor cell lines in xenograft nude mouse models, mediated through surface nucleolin (Destouches et al. 2008; Krust et al. 2011b); HB-19 treatment partly inhibits metastasis of melanoma cells to lymph nodes and lungs (El Khoury et al. 2010). In light of this evidence, it is necessary to look at the dual functions of surface nucleolin as a carcinogenic mediator and as an anti-carcinogenic mediator. This paper reviews the numerous functions of surface nucleolin and the relationship with carcinogenic and anti-carcinogenic ligands. Disease-related ligands that do not belong to the previous two types may show the way of preventing some ailments.

**Table 1:** Ligands of surface nucleolin

Ligands	Sources	Reported function	References
Carcinogenic			
Midkine	Endogenous	Mitogenic, transformation	Kadomatsu and Muramatsu (2004)
Pleiotrophin	Endogenous	Mitogenic, transformation	Kadomatsu and Muramatsu (2004)
Hepatocyte growth factor	Endogenous	Cancer development	Tate et al. (2006)
K-Ras4B	Endogenous	Cancer development	Birchenall-Roberts et al. (2006)
P-selectin	Endogenous	Signal transduction	Reyes-Reyes and Akiyama (2008)
ErbB receptors	Endogenous	Malignant transformation	Di Segni et al. (2008)
Tipα	*H. pylori*	Tumor promotion and progression	Watanabe et al. (2010b)
Anti-carcinogenic			
HB-19	Synthesized	Anti-HIV infection, anti-carcinogenic activity	Nisole et al. (2002)
Lactoferrin	Cow’s milk	Anti-carcinogenic activity	Legrand et al. (2004)
AS1411/GROs	Synthesized	Anti-carcinogenic activity	Ireson and Kelland (2006)
Endostatin	Endogenous	Anti-angiogenic, anti-carcinogenic activities	Shi et al. (2007)
N6L	Synthesized	Anti-carcinogenic activity	Destouches et al. (2011)
Disease-related			
Lipoproteins	Endogenous	Lipid homeostasis	Semenkovich et al. (1990)
Laminin	Endogenous	Neurite outgrowth	Kleinman et al. (1991)
Fructosyllysine	Endogenous	Cell attachment	Krantz et al. (1995)
Group B coxsackievirus	CVB	Viral infection	de Verdugo et al. (1995)
Factor J	Endogenous	Complement inhibitor	Larrucea et al. (1998)
HIV	HIV	Viral infection	Nisole et al. (1999)
Elongation factor Tu	*Francisella tularensis*	Bacterial infection	Barel et al. (2008)
Intimin-γ	*Escherichia coli* O157:H7	Bacterial adherence	Sinclair and O’Brien (2002)
RSV	RSV	Viral infection	Tayyari et al. (2011)
Aβ42	Endogenous	Alzheimer’s disease	Ozawa et al. (2013)

## Specificity of surface nucleolin

The presence of surface nucleolin was first suggested by endogenously phosphorylated proteins on the surface of various cell lines and the phosphoprotein of simian virus 40-transformed mouse fibroblasts (Pfeifle et al. 1981; Pfeifle and Anderer 1983). Surface nucleolin was later identified in vitro in human hepatocellular carcinoma cell line HepG2, and in vivo in angiogenic endothelial cells within the vasculature (Semenkovich et al. 1990; Christian et al. 2003): It is a glycosylated protein found in various cells. Since extranuclear nucleolin undergoes *N*- and *O*-glycosylations, it may be that glycosylation has a role in regulating the function of nucleolin (Carpentier et al. 2005). The *N*-linked glycosylation of cytoplasmic nucleolin is an essential step for the expression of surface nucleolin in various cells (Losfeld et al. 2009). Recently, Hovanessian and his colleagues found that surface nucleolin creates a 500-kDa protein complex that consists of at least eight other proteins: two Wnt-related proteins A and B (WNT7A and WNT7B); Ku80 autoantigen; signal recognition particle (SRP) subunits 68 and 72; receptor for complement component gC1q-R/p32, which lacks N-terminal 72 amino acids; and ribosomal proteins S4 and S6 (Krust et al. 2011a). The features of each protein can be briefly noted as follows: WNT7A and WNT7B are implicated in proliferation and differentiation of both normal and cancer cells (Kirikoshi and Katoh 2002); Ku80 antigen is present on the surface of leukemic and solid tumor cell lines, including T and B lymphomas, myeloid leukemia, neuroblastoma, rhabdomyosarcoma, and breast carcinoma cells (Lynch et al. 2001); SRP68 and 72 are subunits of a ribonucleoprotein complex of 7S RNA and 6 proteins, and SRP is involved in major cellular machinery (Keenan et al. 2001); gC1q-R, also known as hyaluronan-binding protein 1 (HABP 1), plays a significant role in bacterial infections, inflammation, and tumor progression (Bourguignon 2008; Rubinstein et al. 2004); S4 and S6 are components of 40S and 60S ribosomal subunits, which build up ribosomal 80S, and many ribosomal protein genes act as cancer genes in zebra fish (Amsterdam et al. 2004). In addition, ligand binding to surface nucleolin induces high transitory Ca^++^ membrane fluxes in the cells and elevates cell proliferation associated with expression of surface nucleolin levels (Losfeld et al. 2009). All the results show that proliferating cells have surface nucleolin on the plasma membrane and that surface nucleolin forms a large protein complex, but the interaction between the proteins and surface nucleolin has not been clearly established.

## Ligands of surface nucleolin

Ligands are isolated from various sources. To make their functions clearer, they can be classified into three types, carcinogenic, anti-carcinogenic, and disease-related ligands (Table 1). Among carcinogenic ligands, midkine (MK) and pleiotrophin (PTN) are original heparin-binding growth factors, and MK is expressed in a wider rage of human carcinoma cells than PTN is (Kadomatsu and Muramatsu 2004); HGF regulates invasion and growth of carcinoma cells (Tate et al. 2006); K-Ras4B protein is a potent oncoprotein present in the nucleoli of both normal and transformed cells (Birchenall-Roberts et al. 2006); P-selectin binding to human colon carcinoma cell line Colo-320 forms a signaling complex with surface nucleolin (Reyes-Reyes and Akiyama 2008); ErbB receptor tyrosine kinases are major contributors to malignant transformation, and their interaction with nucleolin results in receptor dimerization and anchorage-independent growth (Di Segni et al. 2008); Tipα of *H. pylori*, a carcinogenic factor, will be described in more detail shortly (Watanabe et al. 2010b).

Among anti-carcinogenic ligands, HB-19 pseudopeptide is a potent inhibitor of HIV infection where it acts by blocking virus particle attachment (Nisole et al. 2002); lactoferrin (LF), found in mammalian secretions and blood, inhibits proliferation of cancerous mammary gland epithelial cells and also manifests a potent anti-viral activity against HIV and human cytomegalovirus (Legrand et al. 2004); AS1411, a 26-mer unmodified guanosine-rich oligonucleotide, induces growth inhibition of cancer cells in vitro and shows inhibition of human tumor xenografts in vivo (Ireson and Kelland 2006); guanosine-rich quadruplex-forming oligodeoxy nucleotides (GROs) are anti-carcinogenic ligands (Soundararajan et al. 2009); endostatin (ES), a 20-kDa C-terminal globular domain of the collagen XVIII, was isolated from supernatant of a cultured murine hemangioendothelioma cell line, and it specifically inhibits proliferating endothelial cell growth and angiogenesis (Shi et al. 2007); N6L is a proapoptotic molecule and a synthetic ligand of surface nucleolin that inhibits the anchorage-dependent and anchorage-independent growth of cancer cell lines and angiogenesis (Destouches et al. 2011).

As for disease-related ligands, their functions are varied. Ligands that do not yet show carcinogenic or anti-carcinogenic activities are grouped among the disease-related ligands, derived from various sources: Lipoproteins contained in apoB and apoE bind to surface nucleolin of HepG2 cells, which act as LDL receptors (Semenkovich et al. 1990); laminin is a potent promoter of neurite outgrowth (Kleinman et al. 1991); fructosyllysine is the main glucose adduct in the extracellular proteins formed by a condensation between ε-amino group of lysine and glucose by Amadori rearrangement and is found in human monocytes and monocyte-like cell line U937 (Krantz et al. 1995); group B coxsackieviruses (CVB) are enteroviruses of the family *Picornaviridae* and important human pathogens (de Verdugo et al. 1995); factor J (FJ), a soluble molecule found in urine and serum, is a complement inhibitor that regulates the pathways of a complement (Larrucea et al. 1998); HIV infects CD4^+^ target cells by fusion of the viral and cellular membranes through the external envelope glycoprotein gp120 (Nisole et al. 1999); elongation factor Tu (EF-Tu) of *Francisella tularensis* is a causative agent of tularemia that facilitates invasion of host tissue (Barel et al. 2008); intimin-γ is an outer membrane protein of enterohemorrhagic *Escherichia coli* (EHEC) O157:H7 that colocalizes on the surface of HEp-2 cells with nucleolin (Sinclair and O’Brien 2002); human respiratory syncytial virus (RSV) causes an infectious disease worldwide, and it interacts with host-cell nucleolin through viral fusion envelope glycoprotein (Tayyari et al. 2011); amyloid-β peptide 1-42 (Aβ42) plays a key role in neurotoxicity of Alzheimer’s disease, and it strongly binds to nucleolin, although Aβ40 is weakly bound (Ozawa et al. 2013). In this paper, we will discuss various kinds of ligands and surface nucleolin in focusing on carcinogenic and anti-carcinogenic activities.

## TNF-α as an endogenous tumor promoter

A tumor promoter induces clonal growth of initiated cells, resulting in tumor development. 12-*O*-tetradecanoylphorbol-13-acetate (TPA), teleocidin, and aplysiatoxin—the latter of which are structurally different from TPA—similarly induced potent tumor-promoting activity on mouse skin initiated with 7,12-dimethylbenz[a]anthracene (DMBA), mediated through activation of protein kinase C (Fujiki and Sugimura 1987). Moreover, okadaic acid and calyculin A, which are potent inhibitors of protein phosphatases 1 and 2A, are newly identified tumor promoters as potent as TPA on mouse skin initiated with DMBA (Fujiki and Suganuma 1993). In light of our evidence, we decided to look at endogenous tumor promoters in human carcinogenesis, focusing on the inflammatory cytokines. Our reasons are based on the “inflammation theory” of Rudolf Virchow from Berlin (Virchow 1858) and the carcinogenesis experiment on rabbit ear with coal tar of Katsusaburo Yamagiwa and Koichi Ichikawa in Tokyo (1915; Fujiki 2014). Their contributions led us to our current understanding that the relationship between inflammation and tumor development is strongly associated with up-regulation of NF-κB at the molecular level (Fujiki et al. 2013). We were fortunate to be able to demonstrate that the proinflammatory cytokine TNF-α induces transformation of BALB/3T3 cells initiated with 3-methylcholanthrene (MCA) (Komori et al. 1993), which showed that TNF-α is the essential cytokine in tumor promotion since okadaic acid did not show any tumor-promoting activities in TNF-α-deficient (TNF-α^−/−^) mice initiated with DMBA (Suganuma et al. 1999). Our further experiments revealed that TNF-α is strongly induced in organs of rodents by treatment with various tumor promoters, such as microcystin-LR and nodularin, and that the inflammatory cytokines and chemokines, such as TNF-α, IL-1 and IL-6, are also thought to have tumor-promoting activities in human cancer development (Suganuma et al. 2002; Fujiki and Suganuma 2011). Based on all of these results, we found a tumor promoter gene in *H. pylori* genome that induces *TNF*-*α* gene expression.

## TNF-α-inducing protein (Tipα) of *H. pylori*

**Fig. 1:**
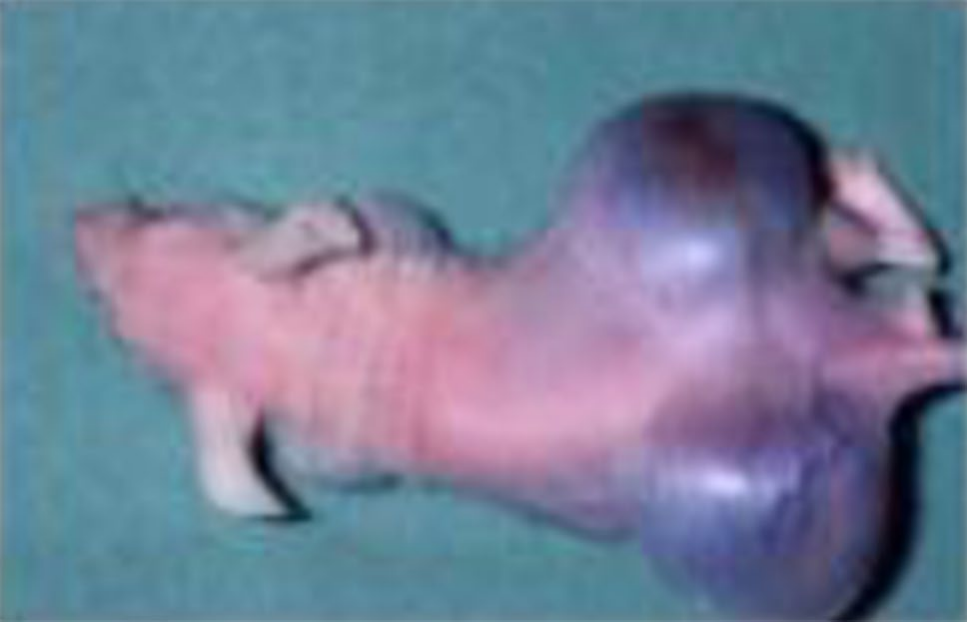
Tumor development of Bhas 42 cells containing *HP*-*MP1* gene in nude mice. Tumor-bearing mouse implanted with Bhas 42 cells containing *HP*-*MP1* gene 28 days later

*Helicobacter pylori* membrane protein 1 (HP-MP1), one of the TNF-α-inducing proteins, has a molecular weight of 16 kDa, and its structure is not related to any virulence factors of *H. pylori* (Yoshida et al. 1999). The transfection of *HP*-*MP1* gene into Bhas 42 cells, the putative initiated cells (v-Ha-*ras*-transfected BALB/3T3 cells), induced *TNF*-*α* gene expression with the levels ranging from 12.2- to 27.0-fold higher than the basal levels of the parental Bhas 42 cells, and the transfected cells induced morphological changes in transformation (Suganuma et al. 2001). Because the Bhas 42 cells containing *HP*-*MP1* gene had shown strong tumorigenicity in nude mice (Fig. 1), we concluded that HP-MP1 protein induced *TNF*-*α* gene expression and TNF-α production in cooperation with v-Ha-*ras* gene product. To generalize the concept of TNF-α-inducing protein as a tumor promoter, we discovered *HP0596* gene, from *H. pylori* strain 26695, in silico from the complete genome sequence (Suganuma et al. 2005). *HP0596* gene was 94.3 % homologous to *HP*-*MP1* gene; the deduced amino acid sequence revealed that HP0596 protein has 172 amino acids with 19 kDa. We therefore designated HP0596 protein as the TNF-α-inducing protein (Tipα): The secreted amount of Tipα was determined using 28 clinical isolates of *H. pylori* obtained from 17 gastric cancer and 11 chronic gastritis patients. Clinical isolates from cancer patients secreted Tipα at 1.4–13.4 relative units, and those from gastritis patients secreted it at 0.8–6.7 relative units, suggesting that Tipα induces cancer development in human stomach infected with *H. pylori*. All tested strains of *H. pylori* secreted both 38-kDa (dimer) and 19-kDa (monomer) proteins in the medium, without dithiothreitol, in a manner independent of type IV secretion system of cagA (Suganuma et al. 2008). To study the molecular mechanisms, we made both recombinant Tipα protein (rTipα) and recombinant Tipα protein 6 amino acids deleted from N-terminal 2-7 (rdel-Tipα) as control, the latter of which lacks two cysteine residues, C5 and C7 (Suganuma et al. 2005).

The quaternary structure of rdel-Tipα showed that rdel-Tipα monomer A interacts with another monomer B, to form a heart-shaped dimer, and their circular dichroism spectra suggested that the structures of rdel-Tipα and Tipα homodimers are very similar (Tsuge et al. 2009). Encouraged by the above-mentioned results, we studied the specific binding protein for Tipα in mouse gastric cancer cell line MGT-40, using fluorescein isothiocyanate (FITC)-labeled rTipα. The specific binding of FITC-rTipα to MGT-40 cells became saturated at a concentration of 5.0–7.5 μM, and this specific binding was inhibited by nonlabeled rTipα (IC_50_ 1.9 μM), less weakly inhibited by rdel-rTipα (IC_50_ 20.0 μM). In addition, treatments of MGT-40 cells with FITC-rTipα at 37 or 4 °C showed that FITC-rTipα internalized into MGT-40 cells in a temperature-dependent manner (Suganuma et al. 2008). The results show that a dimer of rTipα with disulfide bonds binds to a receptor of Tipα on MGT-40 cells and that the receptor-binding complex with rTipα induces *TNF*-*α* and *chemokine* gene expressions in the nuclei of MGT-40 cells (Kuzuhara et al. 2007; Suganuma et al. 2012).

## Surface nucleolin as a receptor for Tipα of *H. pylori*

To identify the specific rTipα-binding protein, we made rTipα-FLAG and rdel-Tipα-FLAG, both of which carry a His-tag in the N-terminal and a FLAG-tag in the C-terminal regions: rTipα-FLAG and rdel-Tipα-FLAG perform the same biological activities as rTipα and rdel-Tipα. After cell lysates of mouse gastric cancer cell line MGT-40 were incubated with rTipα-FLAG or with rdel-Tipα-FLAG in vitro at 4 °C for 2 h, the polypeptides that coimmunoprecipitated with rTipα-FLAG—but not rdel-Tipα-FLAG—were further subjected to tryptic digestion and LC–MS analysis. Since 88- and 40-kDa polypeptides that we obtained showed an identical peptide sequence to that of mouse nucleolin, their polypeptides were further subjected to immunoblot analysis with anti-nucleolin antibody. Pull-down assay using recombinant human nucleolin fragment showed that Tipα directly binds to nucleolin, and additional experiments with cell fractionation and flow cytometry confirmed that the 88-kDa polypeptide is nucleolin, which is located on the cell surface of MGT-40: This was the first evidence that surface nucleolin acts as a Tipα receptor of *H. pylori* (Watanabe et al. 2010b). The results provide a new possibility to study gastric carcinogenesis of *H. pylori* using a nucleolin and Tipα complex.

## Role of surface nucleolin in gastric carcinogenesis in humans

The cell lysates of five human gastric cancer cell lines were divided into membrane, cytosolic, and nuclear fractions, and each fraction was then subjected to SDS–PAGE and further immunoblotted with anti-nucleolin antibody. The amount of surface nucleolin was found to be large in MKN-45 (poorly differentiated adenocarcinoma), KATOIII (signet ring cell carcinoma), MKN-74 (moderately differentiated adenocarcinoma), and AGS (adenocarcinoma) cells; the amount was small in MKN-1 (adenosquamous carcinoma) cells, but all five cell lines contained similar amounts of nucleolin in the whole cell lysates (Watanabe et al. 2010a). In addition, the presence of surface nucleolin was confirmed in all the cell lines by flow cytometry after they were incubated with anti-nucleolin antibody (anti-NUC295) or with preimmune serum in the presence of Alexa Fluor 488-conjugated goat anti-rabbit IgG. It is of interest to note that the amount of surface nucleolin in the normal epithelial membrane fraction of mouse glandular stomach is much lower than that in malignant MGT-40 cells, indicating that translocation of nucleolin to the cell surface occurs during carcinogenesis, making for easier binding to Tipα (Watanabe et al. 2010a). The results are well supported by previous evidence showing that the presence of surface nucleolin in various types of cells is closely associated with cell proliferation (Hovanessian et al. 2000).

The anti-carcinogenic aptamer AS1411, a DNA aptamer of 26-mer unmodified guanosine-rich nucleotide, binds to nucleolin and results in inhibition of nucleolin functions (Ireson and Kelland 2006; Soundararajan et al. 2009). We studied the inhibitory effects of AS1411 on cell growth of five human gastric cancer cell lines, and their IC_50_ values were in the range of 2.3 to >20.0 μM, whereas control cytidine-rich oligonucleotide (CRO) of AS1411 did not show any inhibition. In addition, the incorporation of FITC-AS1411 into the cells of human gastric cancer cell line MKN-45 was detected within 2 h, and the incorporated FITC-AS1411 had colocalized with nucleolin in cytosol and nucleoli (Watanabe et al. 2010a).

**Fig. 2:**
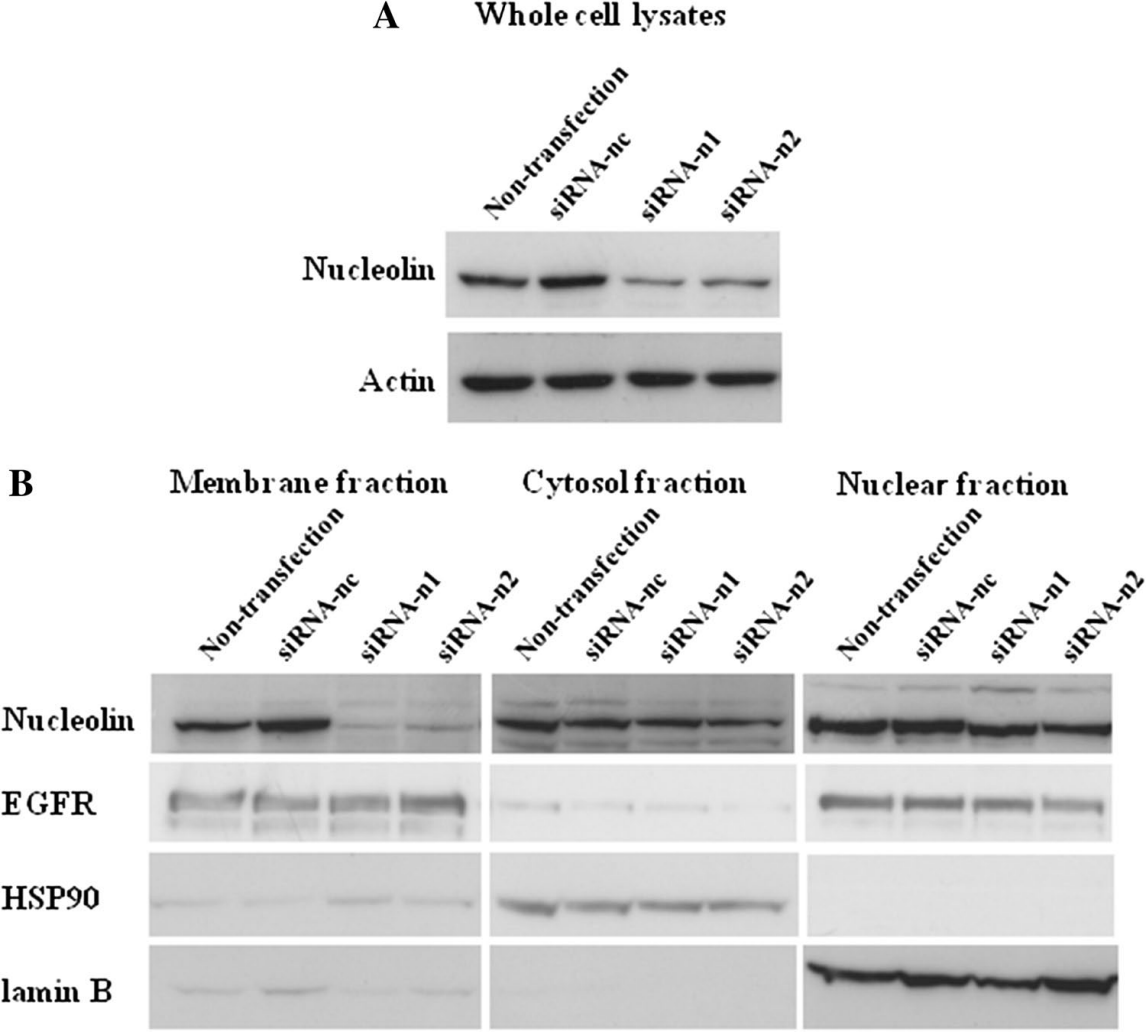
Inhibition of nucleolin expression in MKN-1 cells transfected with nucleolin-targeted siRNAs. **a** Nucleolin expression was determined in whole cell lysates. The cells were treated with nontransfection, siRNA-nc as control, and two nucleolin-targeted siRNAs, siRNA-n1 and siRNA-n2. **b** Nucleolin expression was determined in membrane, cytosol and nuclear fractions in nontransfected and transfected cells, and nucleolin expression was compared with that of EGFR, HSP90, and lamin B proteins

**Fig. 3:**
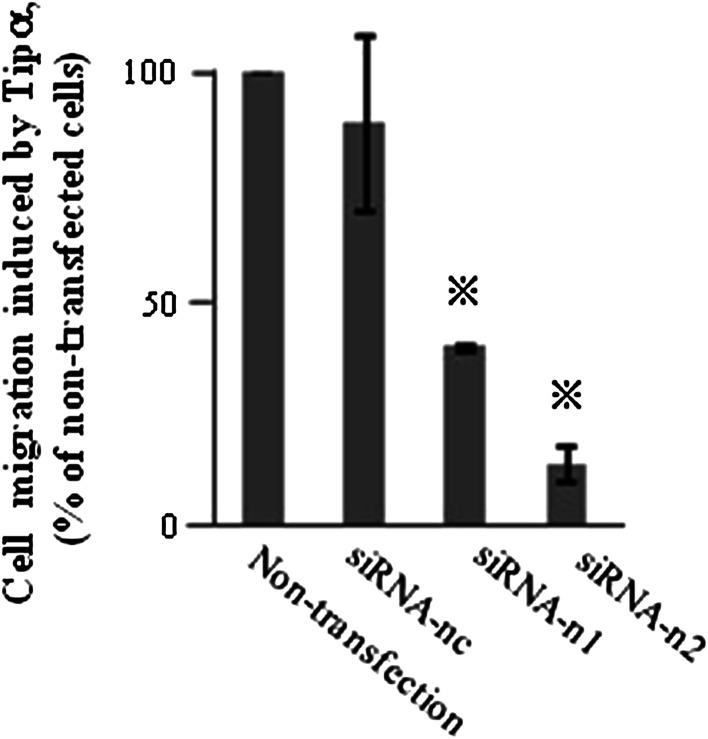
Inhibition of Tipα induced cell migration in MKN-1 cells transfected with nucleolin-targeted siRNAs

To demonstrate the direct involvement of nucleolin in cell migration induced by rTipα, the cells of human gastric cancer cell line MKN-1 were transfected with small interfering RNAs, nucleolin-targeted siRNA-n1 and siRNA-n2. The result was 50 % reduction in the nucleolin in their cell lysates, whereas transfection with negative control siRNA-nc did not reduce nucleolin (Fig. 2a). When cell lysates of the transfected cells were fractionated into membrane, cytosol, and nuclear fractions, transfection with siRNA-n1 and siRNA-n2 resulted in the reduction in nucleolin in the membrane fraction compared with that in the cytosol and nuclear fractions (Fig. 2b). Transfections with siRNA-n1 and siRNA-n2 into MKN-1 cells also inhibited cell migration induced by rTipα 60.4 and 86.1 %, respectively, while inhibition with siRNA-nc as control was marginal (Fig. 3) (Watanabe et al. 2013). All the results showed that transfection with siRNA-n1 and siRNA-n2 significantly inhibited the effects of rTipα, including cell migration, elongation, cell stiffness, and EMT, due to knockdown of surface nucleolin, which indicated that surface nucleolin is directly both a carcinogenic mediator for Tipα and an anti-carcinogenic mediator for AS1411.

## Surface nucleolin on HeLa CD4^+^ cells as a receptor of HIV-1 particle

HIV is an envelope virus that infects target cells by the fusion of viral and cellular membranes. Hovanessian and his colleagues first found that the binding of HIV-1 particle to nucleolin on HeLa CD4^+^ cells was inhibited by two substances: antibody against V3 loop domain of viral external envelope glycoprotein gp120 of HIV-1, which contains a hypervariable region of about 36 amino acids referred to as the V3 loop, and by V3 loop domain-mimicking pseudopeptide 5[K*ψ*(CH2N)PR]-template-assembled synthetic peptide (TASP). The team also obtained the results showing that peudopeptide 5[K*ψ*(CH2N)PR]-TASP (HB-19) inhibits HIV entry into CD4^+^ cells mediated through nucleolin (Callebaut et al. 1998).

**Fig. 4:**
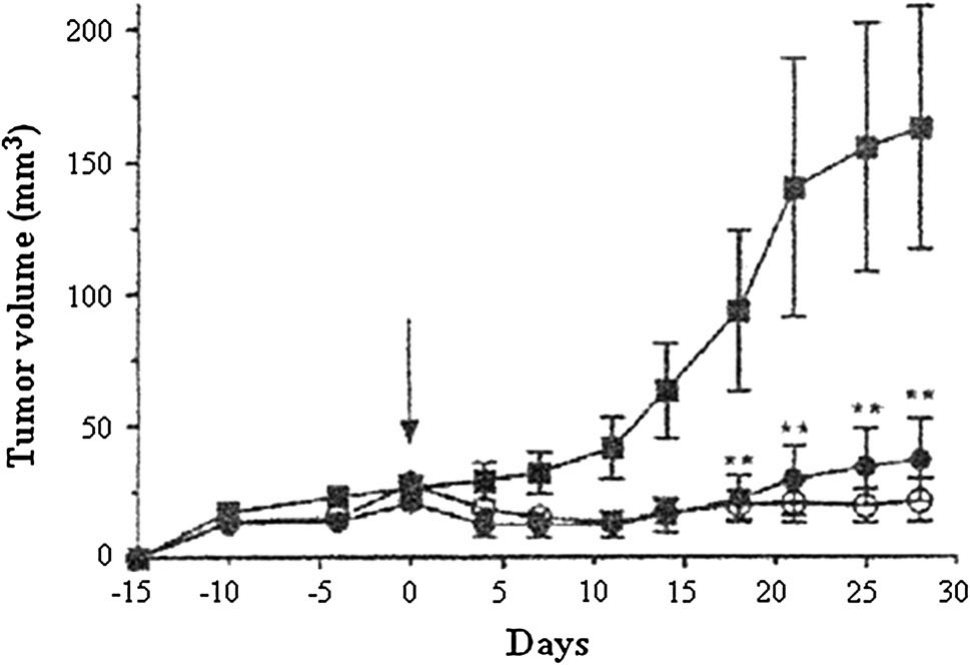
Inhibition of tumor growth of human breast cancer cell line MDA-MB-231 in xenograft nude mouse model treated with PBS alone (*filled square*), HB-19 s.c. (*filled circle*), and HB-19 i.p. (*open circle*)

Based on this evidence, their study was extended to examine inhibitory effects on cell growth and angiogenesis with HB-19, since surface nucleolin is implicated in growth of cancer cells and angiogenesis. When human breast cancer cell line MDA-MB-231 was injected into the mammary fat pad of female nude mice, palpable tumors approximately 40 mm^3^ in volume developed 2 weeks later, and the mice were randomly separated into three groups: The first group was given HB-19 (5 mg/kg) for 6 weeks; the second group was treated with tamoxifen (10 mg/kg) for 6 weeks; and the third group was given PBS only. Treatment with HB-19 reduced tumor weight >95 %, and that with tamoxifen reduced it 80 %. In the next experiments, MDA-MB-231 cell line was inoculated into the flank of female athymic nude mice: Intraperitoneal administrations of HB-19 (i.p., 10 mg/kg) three times per week for a period of 28 days resulted in more than 95 % reduction in tumor volume throughout the experiment, and that of subcutaneous administrations (s.c.) showed similarly strong reduction (Destouches et al. 2008) (Fig. 4). In this carcinogenesis experiment, it is not known which kinds of carcinogenic ligands were produced in MDA-MB-231 cells, but we assume that endogenous inflammatory cytokines and chemokines, which are able to bind to nucleolin, were probably involved in this tumor growth, since tumor promotion and progression are induced by inflammatory proteins (Fujiki et al. 2013). In addition, HB-19 treatment inhibited in vivo angiogenesis in chick embryo chorioallantoic membrane assay, so the results indicate that surface nucleolin itself is also a target for cancer treatment (Destouches et al. 2008).

Anti-carcinogenic activity of HB-19 was next examined using rhabdoid tumor cell line G401 derived from Wilms’ tumor in nude mice. Nontreated G401 cells as control and G401 cells pretreated with HB-19 were separately inoculated into nude mice. The average tumor weight (5 mice) in control group was 2.36 ± 1.25 g at day 50 of the experiment, whereas that of three mice in HB-19-treated group was 0.32 ± 0.18 g (Krust et al. 2011b). The results show that HB-19 treatment inhibited the malignant growth of Wilms’s tumor cells.

## Retardation of melanoma development in RET transgenic mice by HB-19

**Fig. 5:**
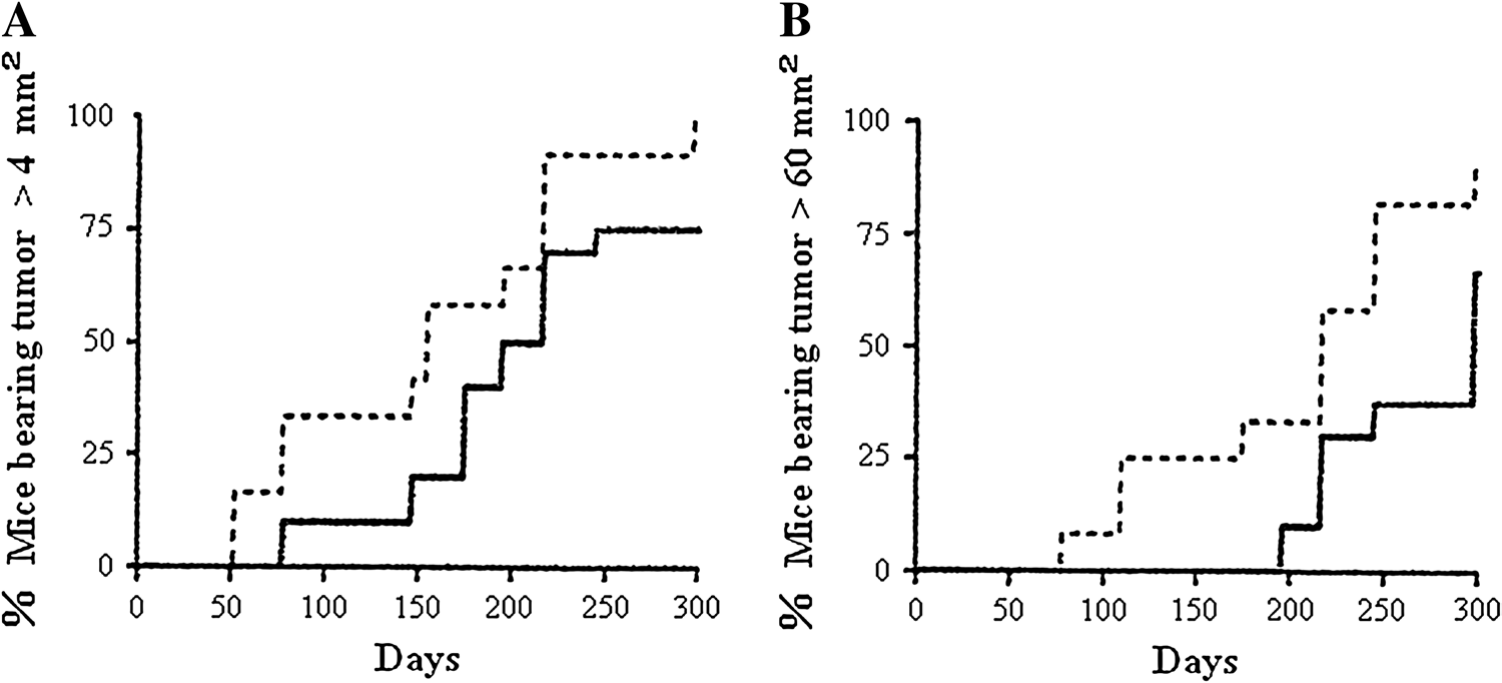
Inhibition of spontaneous melanoma development in RET mice by injections with HB-19. **a** Onset of first melanoma with HB-19 (*dashed line*) and nontreated control (*dotted line*), *p* < 0.001, **b** onset of large melanoma (>60 mm^2^) with HB-19 (*dashed line*) and nontreated control (*dotted line*), *p* < 0.001

MT/ret^+/−^ transgenic mice (RET mice) expressing the *rfp*-*ret* oncogene develop a spontaneous melanoma that constitutively enhances cRET protein expression in the development of melanoma in RET mice (Kato et al. 1998; Ohshima et al. 2010). To study the anti-carcinogenic activity of HB-19, 10-day-old RET mice were treated with 50 μg HB-19, 5 i.p. injections for the first week, 100 μg for the second week, and 200 μg for the rest of the experiment, and control mice received PBS only. The first melanoma in control group appeared at day 50 in the skin, while that in the HB-19-treated group was found at day 75, suggesting that HB-19 treatment delayed the onset of melanoma development (Fig. 5a). When the first appearance of a large melanoma (over 60 mm^2^) was next compared between the two groups, the difference was significant: Large melanoma of control group started to develop from day 75, whereas that of the HB-19-treated group started from day 190, so HB-19 treatment delayed the development of melanoma, but did not eradicate it (Fig. 5b) (El Khoury et al. 2010). Melanoma cells have malignant features in which they metastasize to lymph nodes, mediastinum, and lungs. Eight of 11 control mice (72.7 %) showed either retroperitoneal metastasis or mediastinal adenopathies, whereas only three of nine mice (33.3 %) in the HB-19-treated group showed visceral or lung metastasis: HB-19 treatment partly prevented metastasis of melanoma cells in RET mice. Although the cRET protein is involved in the development of melanoma, it is not yet known whether it binds to surface nucleolin. In addition, the expression of *MMP*-*2*, *MMP*-*9*, and *TNF*-*α* genes is significantly reduced in melanoma recovered from RET mice treated with HB-19: HB-19 seems to have potential for cancer treatment and prevention. The results strongly support the conclusion that surface nucleolin mediates the anti-carcinogenic activity of HB-19, since HB-19 binds to RGG domain at the C-terminal end of nucleolin (El Khoury et al. 2010).

## Discussion

The function of surface nucleolin is now a hot topic in cancer research. Since numerous ligands are derived from various sources (Table 1), this paper discusses the interactions between surface nucleolin and Tipα of *H. pylori* as a model of human gastric carcinogenesis, and also the interaction between surface nucleolin and HB-19, an anti-HIV pseudopeptide, as a model of anti-carcinogenesis. The significance of tumor progression is the induction of EMT phenotypes in human cancer cells, i.e., cancer cells shut down the expression of numerous epithelial cell markers and induce the expression of mesenchymal proteins associated with metastatic states. Based on evidence showing that cells transfected with nucleolin-targeted small interfering RNAs resulted in inhibition of EMT phenotypes, including cell migration and elongation, experiments have clearly demonstrated that the EMT induced by Tipα is mediated through surface nucleolin (Watanabe et al. 2013).

The anti-carcinogenic activity of HB-19 is encouraging, as is that of aptamer AS1411. The binding affinity (*Kα* value) of HB-19 to V3-Bps containing nucleolin is 9.6 × 10^9^ M^−1^, *Kα* value sufficient to inhibit HIV entry to the cells (Callebaut et al. 1998). The results strongly support evidence showing that HB-19 inhibits tumor development in xenograft nude mouse models, and we assume that the mechanisms of anti-carcinogenic activity are dependent on the competitive inhibition by HB-19 and AS1411 of various endogenous inflammatory carcinogenic ligands binding to nucleolin in vivo. Inhibitors of molecular interaction between surface nucleolin and its ligands have recently been reviewed looking at anti-carcinogenic activity (Koutsioumpa and Papadimitriou 2013).

It is remarkable that surface nucleolin binds to numerous ligands, such as DNA and RNA. An NMR study on the interaction of nucleolin with RNA revealed that the first two of four RNA-binding domains (RBDs) of nucleolin are responsible for the binding of nucleolin recognition element (NRE) of RNA molecule, because RBD1, RBD2, and the linker of nucleolin form a molecular clamp: NRE is sandwiched between RBD1 and RBD2 (Allain et al. 2000). If so, we can assume that the molecular clamp receives numerous ligands, including AS1411 (Arumugam et al. 2010).

It is difficult to show precisely how the dual functions of surface nucleolin are determined by ligands, or what the interaction of surface nucleolin with each protein of a nucleolin protein complex is; what we think is that the binding site of surface nucleolin is constantly occupied by endogenous ligands in vivo and that this leads to carcinogenic development in humans. Moreover, the disease-related ligands need to be further investigated, because of significant ailments.

Since studies on the conditional nucleolin knockout cells and inactivation of nucleolin with siRNA in HeLa cells have indicated that nucleolin is absolutely required for proliferation and survival of these cells (Storck et al. 2009; Ugrinova et al. 2007), it is not physiologically possible to completely block the function of surface nucleolin in the cells and in vivo. In order to use surface nucleolin as a therapeutic tool, the chemical and biochemical features of ligands and characterizations of surface nucleolin in target cells need to be further investigated. In summary, surface nucleolin is a carcinogenic, anti-carcinogenic, and disease-related mediator, certainly worth intensive further study.
